# Comparative genomics defines the core genome of the growing N4-like phage genus and identifies N4-like Roseophage specific genes

**DOI:** 10.3389/fmicb.2014.00506

**Published:** 2014-10-10

**Authors:** Jacqueline Z.-M. Chan, Andrew D. Millard, Nicholas H. Mann, Hendrik Schäfer

**Affiliations:** ^1^Oxford Gene TechnologiesBegbroke, UK; ^2^Division of Microbiology and Infection, Warwick Medical School, University of WarwickCoventry, UK; ^3^School of Life Sciences, University of WarwickCoventry, UK

**Keywords:** N4 bacteriophage, Roseobacter, comparative genomics, core genes, auxiliary metabolic genes

## Abstract

Two bacteriophages, RPP1 and RLP1, infecting members of the marine Roseobacter clade were isolated from seawater. Their linear genomes are 74.7 and 74.6 kb and encode 91 and 92 coding DNA sequences, respectively. Around 30% of these are homologous to genes found in Enterobacter phage N4. Comparative genomics of these two new Roseobacter phages and 23 other sequenced N4-like phages (three infecting members of the Roseobacter lineage and 20 infecting other Gammaproteobacteria) revealed that N4-like phages share a core genome of 14 genes responsible for control of gene expression, replication and virion proteins. Phylogenetic analysis of these genes placed the five N4-like roseophages (RN4) into a distinct subclade. Analysis of the RN4 phage genomes revealed they share a further 19 genes of which nine are found exclusively in RN4 phages and four appear to have been acquired from their bacterial hosts. Proteomic analysis of the RPP1 and RLP1 virions identified a second structural module present in the RN4 phages similar to that found in the *Pseudomonas* N4-like phage LIT1. Searches of various metagenomic databases, including the GOS database, using CDS sequences from RPP1 suggests these phages are widely distributed in marine environments in particular in the open ocean environment.

## Introduction

Phages (viruses that infect bacteria) are the most prevalent entities in the biosphere; they harbor a vast, untapped reservoir of genomic diversity and are important in driving the evolution of bacteria (Rohwer, 2003; Paul and Sullivan, 2005; Angly et al., 2006). They are also a significant component of the microbial food web and have major influence on fluxes of organic and inorganic matter, in particular in the oceans (Fuhrman, 1999; Wilhelm and Suttle, 1999; Weinbauer and Rassoulzadegan, 2004; Suttle, 2005, 2007; Breitbart et al., 2007). Metagenomic surveys suggest that the true diversity of marine phages exceeds that represented by isolated phages (Breitbart and Rohwer, 2005; Angly et al., 2006; Hurwitz and Sullivan, 2013) and there remain major gaps in understanding which hosts are infected by the wide diversity of phage observed in the environment.

One of the major groups of bacteria found in the marine environment is the so-called Roseobacter clade. Its members represent a taxonomically and metabolically diverse group of bacteria found in pelagic and benthic habitats where they play key roles in a wide range of biogeochemically important transformations (Buchan et al., 2005). Processes affecting their abundance and activity, such as viral lysis, are of biogeochemical significance but are currently poorly understood as only a small number of bacteriophages interacting with Roseobacters (roseophages) have previously been described. The first isolated roseophage was SIO1 (Rohwer et al., 2000), but since then four lytic roseophages infecting *Roseobacter denitrificans* (phage RDJLΦ 1), *Ruegeria pomeroyi* (phage DSS3Φ2), *Sulfitobacter* strain EE36 (phage EE36Φ1) and *Sulfitobacter* strain 2047 (phage pCB2047-B) have been described (Zhang and Jiao, 2009; Zhao et al., 2009; Ankrah and Budinoff, 2014). The latter three are closely related to Enterobacteria phage N4, which, for over 40 years, was the sole representative of the N4-like genus, a genetic orphan among the tailed phages (Schito et al., 1965; Ceyssens et al., 2010). N4 was unique in the phage world due to its use of three distinct RNA polymerases and single-stranded DNA protein/activators to control gene expression (Choi et al., 2008). In recent years a further 25 N4-like phages have been isolated and genome sequenced (Table 1) all of which share these features.

**Table 1:** **N4-like bacteriophages for which genome sequences are available**.

**Phage**	**Host**	**Isolation location**	**Genome size (kb)**	**Accession number**	**References**
N4	*Escherichia coli* K12	Sewage water, Genoa, Italy	70.2	EF056009	Schito et al., 1967
DSS3Φ2	*Ruegeria pomeroyi* DSS-3	Baltimore Inner Harbor water, USA	74.6	FJ591093	Zhao et al., 2009
EE36Φ1	*Sulfitobacter* sp. EE-36	Baltimore Inner Harbor water, USA	73.3	FJ591094	Zhao et al., 2009
LIT1	*Pseudomonas aeruginosa* US449	Belgian hospital sewage, Belgium	72.5	NC_013692	Ceyssens et al., 2010
LUZ7	*Pseudomonas aeruginosa* Br257	Belgian hospital sewage, Belgium	74.9	NC_013691	Ceyssens et al., 2010
PEV2	*Pseudomonas aeruginosa* PAV237	Sewage water, Olympia, WA, USA	72.7	n/a	Ceyssens et al., 2010
S6	*Erwinia amylovora*	Fruit production environment, Switzerland	74.7	HQ728266	Born et al., 2011
KBNP21	*Escherichia coli* KBP21	Chicken farm, in Yesan, South Korea	69.9	JX415535	Nho et al., 2012
PA26	*Pseudomonas aeruginosa* ATCC 27853	Reservoir water, Naju City, South Korea	72.3	JX194238	Kim et al., 2012
G7C	*Escherichia coli* strain 4s	Horse feces	71.8	HQ259105	Kulikov et al., 2012
IME11	*Escherichia coli*	Sewage of the no. 307 hospital in Beijing, China	72.6	JX880034	Fan et al., 2012
EC1-UPM	*Escherichia coli* O78:K80	Chicken feces	70.9	KC206276	Gan et al., 2013
FSL SP-058	*Salmonella* serova Dublin	Dairy farm	72	KC139517	Moreno Switt et al., 2013
FSL SP-076	*Salmonella* serova Dublin	Dairy farm	72	KC139520	Moreno Switt et al., 2013
JA1	*Vibrio cholerae* O139	Stool of a Vibrio cholerae O139 Bengal-infected patient	69.3	KC438282	Fouts et al., 2013
Presely	*Acinetobacter baumannii* M2	sewage sample collected in College Station, TX, USA	77.2	KF669658	Farmer et al., 2013
VCO139	*Vibrio cholerae* O139 Bengal	Sewage effluent from the International Centre for Diarrheal Disease Research, Bangladesh	68.9	KC438283	Fouts et al., 2013
JW Alpha	*Achromobacter xylosoxidans* DSM 11852	Waste water treatment plant in Werl, Germany	72.3	KF787095	Wittmann et al., 2014
JW Delta	*Achromobacter xylosoxidans* DSM 11852	Waste water treatment plant in Braunschweig, Germany	73.7	KF787094	Wittmann et al., 2014
pCB2047-B	*Sulfitobacter* sp. strain 2047	Mesocosm study, Raunefjorden, Norway	74.5	HQ317387	Ankrah and Budinoff, 2014
EcP1	*Escherichia coli* strain 285	Hospital raw sewage, China	59.1	HQ641380	unpublished
pYD6-A	*Pseudoalteromonas* sp. YD6	Surface coastal water, South China Sea	76.8	NC_020849	unpublished
VBP32	*Vibrio parahaemolyticus* RIMD2210633	Lobster Hatchery Stonington, ME	76.7	HQ634196	unpublished
VCP47	*Vibrio parahaemolyticus* RIMD2210633	Lobster Hatchery Stonington, ME, USA	76.7	HQ634194	unpublished
RLP1	*Roseovarius* sp. 217	Langstone Harbour, Hampshire, UK	74.6	FR682616	This study
RPP1	*Roseovarius nubinhibens*	L4 sampling station, Plymouth, UK	74.7	FR719956	This study

The aim of this study was to isolate and characterize lytic phages infecting members of the Roseobacter clade using a number of different Roseobacter host strains and samples of coastal seawater from the United Kingdom. We isolated two new Roseobacter N4-like phages (RN4-phages) that infect *Roseovarius nubinhibens* and *Roseovarius* sp. 217. Here, we report the sequencing of their genomes and the identification of phage-particle associated proteins by mass spectrometry. With the increased number of genome sequences available for N4-like phages it was possible to address questions regarding the structure and evolution of the genomes of this growing group of phages.

## Materials and methods

### Growth of bacterial strains

Cultures of *Rsv. nubinhibens* (Gonzalez et al., 2003) and *Rsv.* sp. 217 (Schäfer et al., 2005) were routinely grown in Marine Ammonium Mineral Salts amended with 10 g L^−1^ peptone and 5 g L^−1^ yeast extract (MAMS-PY).

### Phage isolation

Phages were isolated from seawater samples collected from the English Channel at the L4 sampling station situated approx. 10 nautical miles south of Plymouth, Devon, UK, 50°15′N, 04°13′W (http://www.westernchannelobservatory.org.uk/) on 24-11-1998 and Langstone Harbour on 17-09-2005 (Hampshire, UK). Seawater samples, supplemented with Yeast/Peptone (1 g L^−1^/5 g L^−1^ respectively), were inoculated with *Ruegeria* sp. 198, *Rhodobacteraceae* bacterium 176, *Rsv. nubinhibens*. *Rsv.* sp. 257 and *Rsv.* sp. 217 (Gonzalez et al., 2003; Schäfer et al., 2005) to enrich any Roseobacter phages present. After incubation for 7 days, cells and large cellular debris were removed by centrifugation and the supernatant used in plaque assays against the species in the original inoculum. Clear plaques could be observed on bacterial lawns of *Rsv. nubinhibens* and *Rsv* sp. 217 after 24–48 h incubation at 25°C. The plaques were then picked and made clonal.

### Production of phage stocks

The clonal phage samples made from agar plugs were used in plaque assays to produce plates with confluent lysis of the *Roseovarius* lawn. The top agar layer was removed using a flame-sterilized glass microscope slide and mixed with 3 ml (per plate) of artificial seawater (ASW) modified as described in Wilson et al. (1996). Chloroform was added to a final concentration of 25% (v/v) to lyse remaining host cells. The resulting slurry was mixed thoroughly for at least 1 min and incubated for at least 30 min at room temperature in the dark. The top agar and chloroform was removed by centrifugation at 1780 × *g* for 10 min at 4°C. This typically produced stocks of 1 × 10^8^ plaque-forming units (PFU) ml^−1^. Phages were further purified using CsCl gradient centrifugation for subsequent electron microscopy, DNA extraction and virion proteomic analyses (Sambrook and Russell, 2001).

### Modified bacteriophage one-step growth curve

Bacterial host cells grown in MAMS-PY in early exponential phase were harvested by centrifugation (4000 rpm/1300 × *g*, 15°C for 10 min). The cells were then washed in Marine Broth (Pronadisa, Conda, Madrid) and centrifuged again at 16000 × *g* at room temperature for 10 min. The pellet was resuspended in sterile Marine Broth containing enough phage to have a multiplicity of infection of 0.001. Prior to addition of bacterial host cells, aliquots of the Marine Broth + phage solution had been removed to act as control samples. Both “bacteria + phage” and “phage-only” samples were then plated using the top agar overlay technique and the time noted for each plate. The plates were then transferred to a dark, 20°C incubator for the duration of the experiment.

At appropriate intervals plates were removed and the top agar layer removed with a flame-sterilized glass slide. This was mixed with 3 ml ASW and 3 ml chloroform or cold 3 ml ASW. The period of time between plating and mixing with the ASW:chloroform or cold ASW only solution was taken as time of incubation.

All samples were left at 4°C in the dark overnight then centrifuged at 1300 × *g* at 4°C for 10 min to separate the agar and chloroform. The number of free plaque forming units in the supernatant was then analyzed by appropriate dilution and plaque assays. Each time point for bacterial/phage samples was assayed in triplicate, control samples in duplicate and each growth curve was repeated three times.

### Phage genomic DNA digestion with *Bal31*

CsCl-purified phage stocks were dialysed twice using size 3/MWCO 12-14,000 Da, dialysis tubing for at least 2 h in ASW at 4°C. DNA was isolated and purified using a phenol-chloroform extraction as described previously (Sambrook and Russell, 2001). To determine the physical structure of the genome of the two phages (linear or circular), around 40 μg of phage DNA was digested with *Bal31* at 30°C as described elsewhere (Loessner et al., 2000). Briefly, samples were removed 0, 5, 10, 20, 40, and 60 min after the addition of the enzyme and the digest stopped by incubation at 65°C for 10 min. All samples were purified by phenol-chloroform extraction, precipitated with sodium acetate and ethanol which was followed by digestion with *Nde*1 fast digest (Fermentas) according to manufacturer's instructions. The digest patterns were analyzed by pulsed field gel electrophoresis using a 1% PFGE grade agarose gel run in a CHEF Mapper (BioRad).

### Phage genome sequencing

RLP1 and RPP1 phage DNA was extracted from CsCl stocks and dissolved in 10 mM Tris 1 mM EDTA buffer pH 8 (TE). The genomes were sequenced by the GenePool at the University of Edinburgh using Illumina for RPP1 and a combination of Illumina and Roche 454 shotgun sequencing for RLP1. Short-read Illumina data from RPP1 were assembled using Velvet (Zerbino and Birney, 2008), whereas the mixture of 454 and Illumina reads from RLP1 was assembled using Minimus (Sommer et al., 2007).

RPP1 assembled into a single contig whilst RLP1 assembled into 10 contigs; initial annotation of the largest contig suggested a high degree of gene synteny between RLP1 and RPP1. Consequently, RPP1 was used as a scaffold for RLP1 and the order of contigs was confirmed by PCR. Sequencing of the PCR products (by Sanger sequencing) resulted in complete assembly of RLP1. Whole-genome sequence data was submitted to EBML under accession numbers FR682616 and FR719956 for RLP1 and RPP1 respectively.

### Identification of coding sequences

Coding sequences (CDSs) were predicted using the freely available gene prediction programs GeneMark™, heuristic approach (Besemer and Borodovsky, 1999) and GLIMMER 3.01 (NCBI) (Delcher et al., 1999). The final set of predicted CDSs for each genome was created by amalgamation of the two sets of results from GeneMark and GLIMMER. For predicted CDSs with discordant start codons between the two programs, the longer of the two predictions was kept.

### Database searches

Basic Local Alignment Search Tool (BLAST) comparisons were carried out on the predicted CDSs using different custom-made databases (Altschul et al., 1990). Initially, a search using the BLASTp algorithm of the predicted protein sequences from the two *Roseovarius* phages to a database containing all bacteriophage protein sequences freely available in July 2008 was performed. This was then repeated using BLASTp against the non-redundant protein sequences database at the National Centre for Biotechnology Information (NCBI). In addition, HMMER was used to search the SWISS-PROT database. The results from the three searches were compared to assign putative function to each predicted CDS in RLP1 and RPP1.

To examine the environmental distribution of RN4 phages CDS sequences from RPP1 were used as query sequences for the BLAST algorithm against the environmental metagenomes downloaded from CAMERA (accession numbers CAM_PROJ_HumanGut, CAM_PROJ_AntarcticAquatic, CAM_PROJ_BotanyBay, CAM_P_0000545, CAM_P_0000915, CAM_PROJ_GOS, CAM_PROJ_SalternMetagenome) and EBI for metagenomes from freshwater lakes Bourget (MET6) and Pavin (MET7) (accession ERS015568 and ERS015567 respectively). tBLASTx analysis was carried out with the following parameters modified from default settings –F F –b 100000 –v 100000 –e 0.0001. A reciprocal blastp analysis was then carried out against a custom database of viral sequences. This was constructed from all complete viral genomes available from http://ftp.ncbi.nlm.nih.gov/genomes/Viruses as of February 2013. RPP1 was chosen as a representative of RN4 phages as it has the same complement of genes as RLP1, and an additional three genes. A sequence identified in a metagenome was only considered to be of RN4-like origin if RPP1 was one the top four results in a BLAST search against the viral database described above. The top four were considered as there is significant similarity between the proteins of the RN4 phages DSS3Φ2, EE36Φ1, RLP1 and RPP1 that were also in the blast database.

To account for the difference in size between genes and between metagenomic libraries a similar approach to that taken by Zhao et al. was employed (Zhao et al., 2013). The number of hits for each gene was divided by the number of sequences in the database, this was then divided by the size of the gene product. Samples were then scaled using the mean of all samples, to reduce the number of significant figures. Counts are presented as normalized relative abundance of each gene.

To determine how RN4 phage abundance changes within the defined environmental sites of the Global Ocean Survey (Venter et al., 2004) the same approach was carried out for individual sampling station using ORFs 24, 36, and 51 (the three most abundant ORFs in the eight metagenome examined) as queries.

### CDS/genome comparisons

Phage genome comparisons of all the available N4-like phages were carried out using Orthomcl (Li et al., 2003) which computes a bidirectional best hit search in the amino acid space (with an e-value Cutoff −1e^−06^, *I* = 1.5). The initial database was constructed of the amino acid sequence of all predicted proteins extracted from publically available files in Genbank.

### Phylogenetic analyses

The evolutionary history of selected genes encoding thioredoxins and the core N4-like genome was inferred using the Neighbor-Joining method (Saitou and Nei, 1987). The bootstrap consensus tree inferred from 1000 replicates was taken to represent the evolutionary history of the taxa analyzed (Felsenstein, 1985). Branches corresponding to partitions reproduced in less than 50% bootstrap replicates were collapsed. The evolutionary distances were computed using the Poisson correction method (Zuckerkandl and Pauling, 1965) and all positions containing gaps and missing data were eliminated from the dataset. Phylogenetic analyses were conducted in MEGA5 (Tamura et al., 2007).

### Extraction of phage structural proteins and sodium-dodecyl-sulfate polyacrylamide gel electrophoresis

High titre suspensions of RLP1 and RPP1 roseophage stocks were purified twice on a CsCl step gradient to remove host cellular protein contaminants. 0.01 volume of 2% (w/v) sodium deoxycholate was added to the phage sample and left on ice for 30 min. Trichloracetic acid was added to the samples to a final concentration of 12% (w/v) and the sample was left on ice for 30 min. The precipitated proteins were harvested by centrifugation using a TLA-100.3 (Beckman Coulter) at 37200 × *g* at 4°C for 20 min. The pellet was washed twice in cold acetone then left to air dry. The dry pellet was re-suspended in 1 × Laemmli buffer (50 mM Tris-HCl pH 6.8, 2% (w/v) SDS, 10% (v/v) glycerol, 1% (v/v) β —mercaptoethanol, 12.5 mM EDTA, 0.02% (w/v) bromophenol blue). All samples were denatured at 100°C for 10 min prior to electrophoresis on a 10–20% sodium dodecylsulfate (SDS) gradient polyacrylamide gel using a dual slab gel kit (C.B.S. Scientific) run overnight at 100 V. Protein bands were visualized using Coomassie stain.

### Mass spectrometry analysis of phage proteins

Protein bands of interest were excised from SDS-PAGE gels and tryptically digested using the manufacturer's recommended protocol on the MassPrep robotic protein handling system (Waters). The extracted peptides from each sample were analyzed by means of nanoLC-ESI-MS/MS using the NanoAcquity/Q-ToFUltima Global instrumentation (Waters) using a 45-min LC gradient. All MS data were corrected for mass drift using reference data collected from the [Glu^1^]-Fibrinopeptide B (human—F3261 Sigma) sampled each minute of data collection. The data were then used to interrogate a database made up of the predicted protein sequences from RLP1 or RPP1 appended with the **c**ommon **R**epository of **A**dventitious **P**roteins sequences (http://www.thegpm.org/cRAP/index.html) using ProteinLynx Global Server v2.3. All protein identification was carried out in the in-house Biological Mass Spectrometry and Proteomics Facility of the School of Life Sciences at the University of Warwick.

## Results and discussion

### Isolation and characterization of phages RPP1 and RLP1

Two lytic phages RLP1 and RPP1, infecting two strains of *Roseovarius* were isolated from seawater collected from Langstone Harbour, Hampshire, UK and from water collected from station L4 in the English Channel, respectively. The phages were named using the nomenclature suggested by Kropinski et al. (2009); vB_Rsv217_RLP1 (RLP1, Roseovarius Langstone Podovirus) which infects *Roseovarius* (*Rsv.*) 217 (Schäfer et al., 2005) and vB_RsvN_RPP1 (RPP1, Roseovarius Plymouth Podovirus) which infects *Rsv. nubinhibens* (Gonzalez et al., 2003).

The phages did not infect a number of other Roseobacter group isolates tested including *Rsv. crassostreae*. *Rsv. mucosus*. *Ruegeria pomeroyi* DSS-3, *Ruegeria atlantica*. *Marinovum algicola*. *Sagittula stellata* E-37, *Leisingera methylohalidivorans* MB2, *Rhodobacteraceae* bacterium 176, and *Ruegeria* sp. 198. The susceptible hosts for which phage were isolated, *Rsv. nubinibens* and *Rsv.* sp 217, are 93.5% identical in their 16S rRNA genes but the phage isolated from *Rsv. nubinhibens* was not able to lyse *Rsv.* sp. 217 and vice versa. Based on pairwise 16S rRNA identity the strain most closely related to *Rsv.* sp. 217 is *Rsv. mucosus* with 99% sequence identity, but that strain was not lysed by RLP1 either, demonstrating a very narrow host range of phage RLP1. Interestingly, such a narrow host range has also been observed with other N4-like phages (Zhao et al., 2009; Ceyssens et al., 2010; Kulikov et al., 2012; Fouts et al., 2013) and appears to be a property of many podoviruses (Sullivan et al., 2003; Hess, 2008).

**Figure 1:**
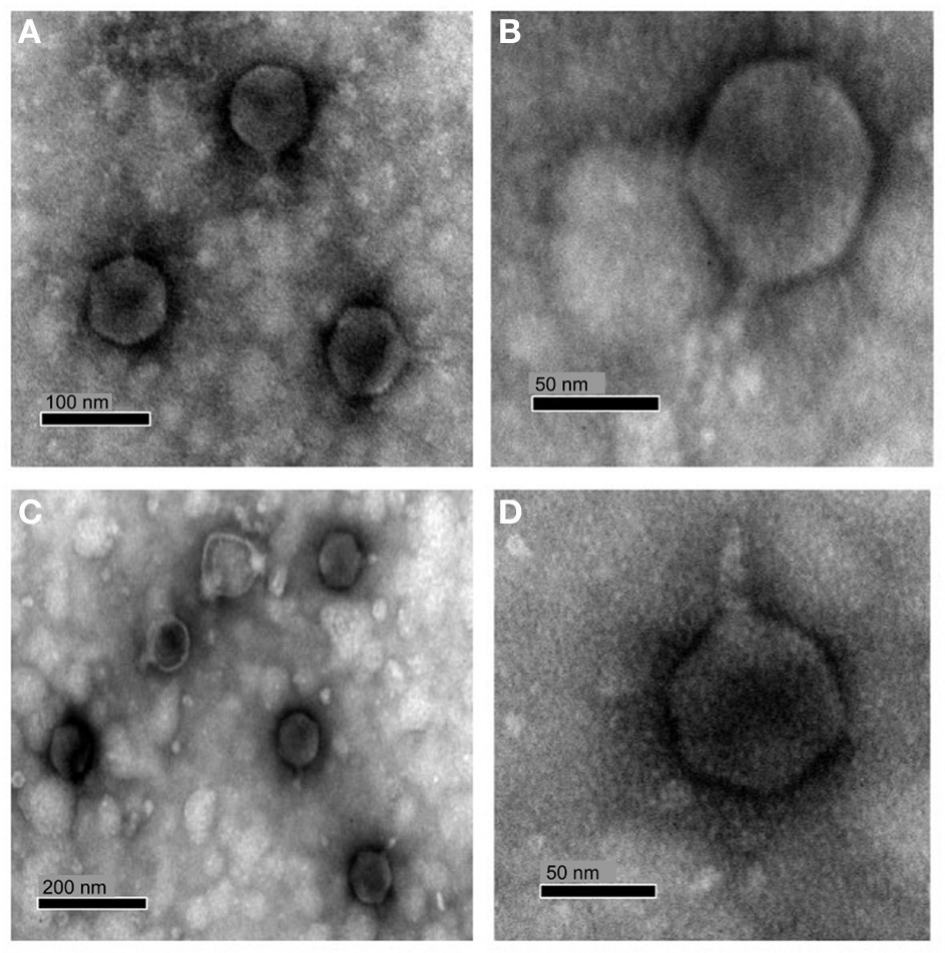
**TEM micrograph of RLP1 and RPP1 negatively stained with uranyl acetate**. RLP1—**(A,B)** and RPP1—**(C,D)**. Based on their morphology phages were classified into the *Podoviridae* family. Magnification: **(A)** × 120,000, **(B)** × 300,000 **(C)** × 75,000 and **(D)** × 200,000.

Infection using soft agar overlays with both phages produced clear plaques around 0.5–2 mm in diameter after *ca*. 48 h incubation with susceptible hosts and infectivity was found to be unaffected by chloroform treatment. Transmission electron microscopy (TEM) of purified virions revealed phages with icosahedral heads and short tails (Figure 1), characteristics typical of the family *Podoviridae*. RLP1 and RPP1 had capsid head sizes of 72.4 ± 2 and 77.4 ± 5 nm respectively.

### Host-virus interactions

**Figure 2:**
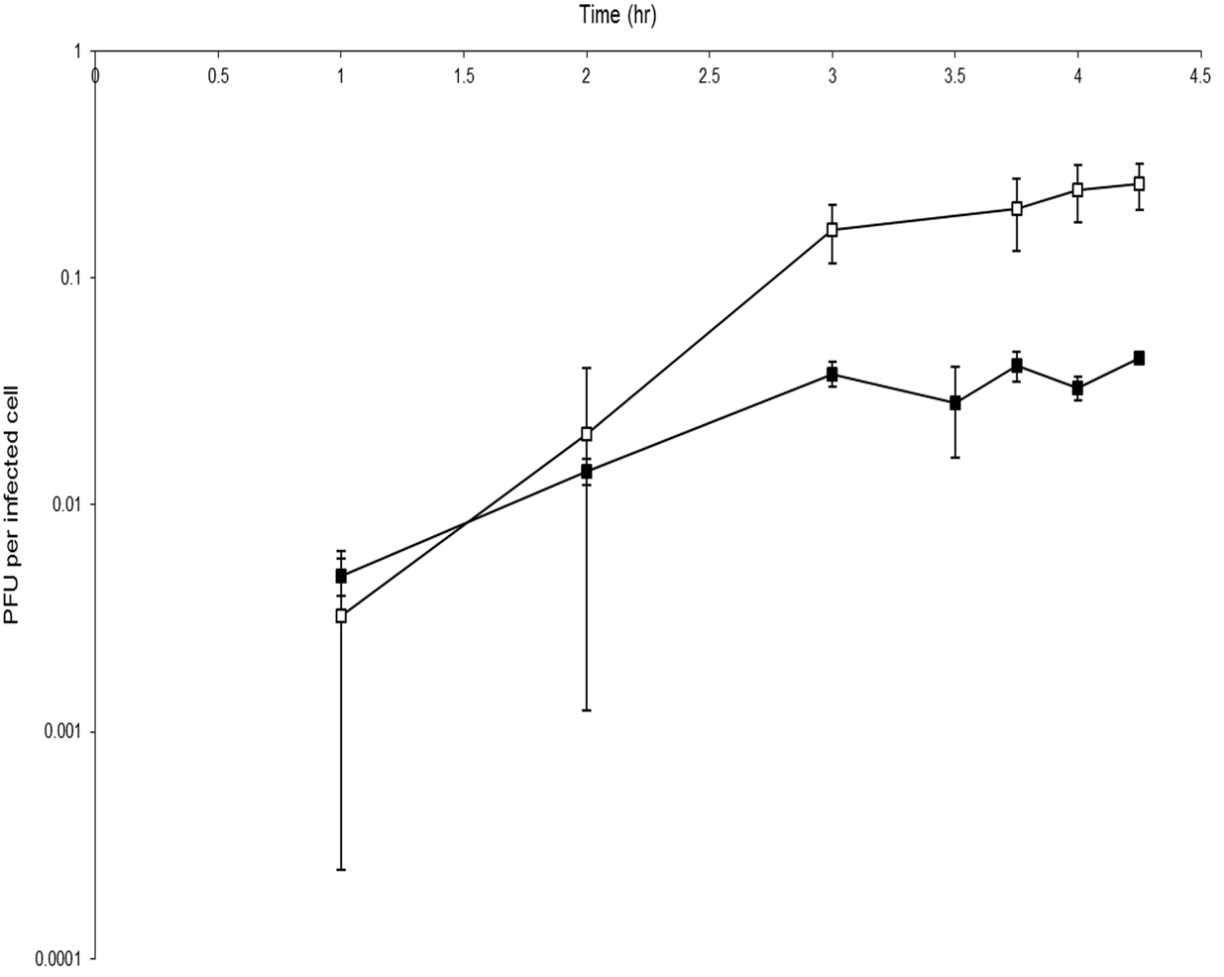
**Modified one-step growth cure for phages RLP1 and RPP1**. Host cells were infected with a MOI of 0.001. One step growth curve of RLP1 on Rsv. 217 (□) and RPP1 on *Rsv. nubinhibens* (■). The number of phage increases over time indicating infection has occurred. There is a marked increase in phage between 2 and 3 h which suggests a burst event has occurred during this period. Each growth curve was performed in triplicate.

In laboratory conditions RLP1 and RPP1 only infected host cells when in semi-solid agar matrix, but not in liquid culture. Therefore, it was not possible to carry out a standard liquid-based one-step growth curve analysis and a modified assay was performed using infected hosts embedded in double-layer agar plates in order to characterize some basic properties of these phages (see Materials and Methods for details). In the modified assay, immediate processing of samples taken during infection (to determine nascent and mature/free phage) was not possible as both infected and un-infected host cells and nascent and mature/free phages were trapped within the top agar matrix and therefore not available for plaque assay. Instead an additional overnight incubation of the top agar layer in phage buffer, to allow diffusion of phage particles out of the matrix, was required prior to enumeration. To quench phage replication mid-cycle, chloroform was added to the phage buffer. As a result only the total plaque forming units (PFU), comprised of both nascent and mature phage, could be determined. The results suggest that the eclipse period for both phages is between 2 and 3 h and the latent period is between 4 and 6 h (Figure 2), however, without a free phage infection profile this cannot be verified. RLP1 appears to have a larger burst size compared to that of RPP1, ~100 PFU cell^−1^ and ~10 PFU cell^−1^, respectively. A precise number for burst size could not be calculated as it is likely that the infected cells were not synchronized and it is possible that multiple infections of a single bacterium occurred as infected cells were not diluted as occurs in a standard one-step growth assay. Compared to EE36Φ 1 and DSS3Φ 2, which had latent periods of 2 and 3 h respectively, the phages obtained here had slightly longer latent periods although data have to be interpreted with caution due to the use of a modified one-step experiment.

### Genome sequence and structure of phages RPP1 and RLP1

The genome sizes of phages RPP1 and RLP1 determined by whole-genome sequencing were 74.7 and 74.6 kb, respectively, which was in good agreement with estimates based on PFGE (Supplementary Material Figure 1). Both phages have a GC content of 49% in contrast to their hosts, *Roseovarius* sp. 217 and *Rsv. nubinhibens*, which have a GC content of 60 and 63%, respectively.

**Figure 3:**
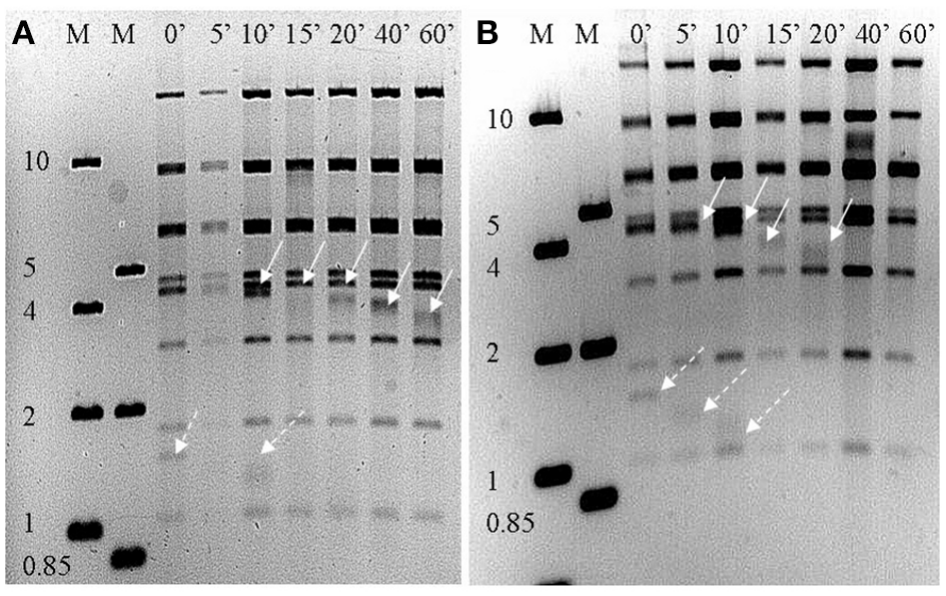
***Nde1* digested (A) RLP1 and (B) RPP1 genomic DNA after treatment with *Bal31* for the indicated time intervals**. Solid arrows indicate restriction fragment decreasing over time, dotted arrows indicate possible second disappearing restriction fragment. The presence of fragments reducing in size with time indicates the phage genome is linear not circular. M, DNA marker (kb).

**Figure 4:**
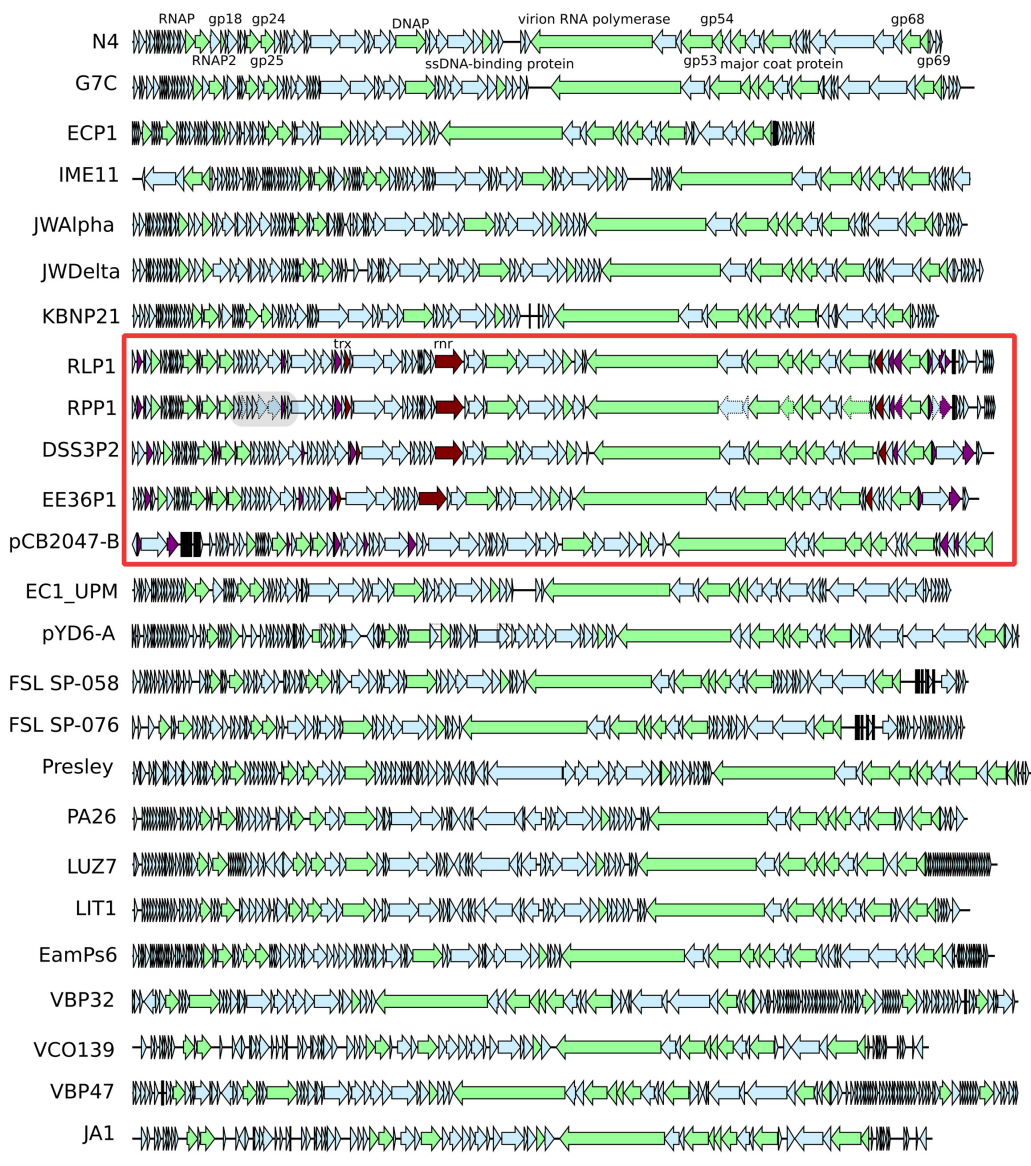
**Comparison of 25 N4-like phage genomes**. Arrows represent the predicted ORFs and point in the direction of transcription. N4-like core genes are shaded in green and labeled with N4 phage homolog ORF numbers, host-like genes found in Roseobacter N4-like phages are shaded in red, and finally experimentally determined structural genes are outlined by dotted lines. The gray box in RPP1 marks the putative second structural module containing experimentally identified virion proteins. The genomes of RLP1 and RPP1 were deposited with EMBL under accession numbers FR682616 and FR719956, respectively.

Both phage genomes were determined to be linear dsDNA through *Bal31*/*Nde1* double digest treatment (Figure 3). The presence of two progressively shortening bands is indicative of a linear genome with defined ends. Gene prediction identified 92 and 91 putative CDSs in RLP1 and RPP1 respectively. Most CDSs (in both phages) appear to initiate at an ATG codon although around 10% use GTG or TTG as start codons. Three transfer RNA genes were also identified in both phages for proline (CCA), isoleucine (ATC) and glutamine (CAA). The two *Roseovarius* phages are highly related in almost all putative CDSs; RLP1 has only three unique CDSs (gps 61, 83, 84) and RPP1 also has three (gps 2, 3, 83) all of which have unknown function. At the nucleotide level, gene homologs are 95–100% similar. Sequence comparison of the two phage genomes demonstrated that there are no large-scale genomic re-arrangements. Overall, the genome structures of RPP1 and RLP1 are similar to those of RN4-phages DSS3Φ 2 and EE36Φ 1 but different to that of pCB2047-B (Zhao et al., 2009; Ankrah and Budinoff, 2014) (Figure 4).

Twenty-eight (~30%) of the predicted CDSs in RLP1/RPP1 are related to those found in Enterobacteria phage N4 and a further 19 CDSs are similar to genes found in roseophages DSS3Φ 2, EE36Φ 1 and pCB2047-B (Table 2). Unlike N4 and N4-like *Pseudomonas* phages no promoter consensus sequences could be identified to assign the predicted CDSs to early, middle or late genes.

**Table 2:**
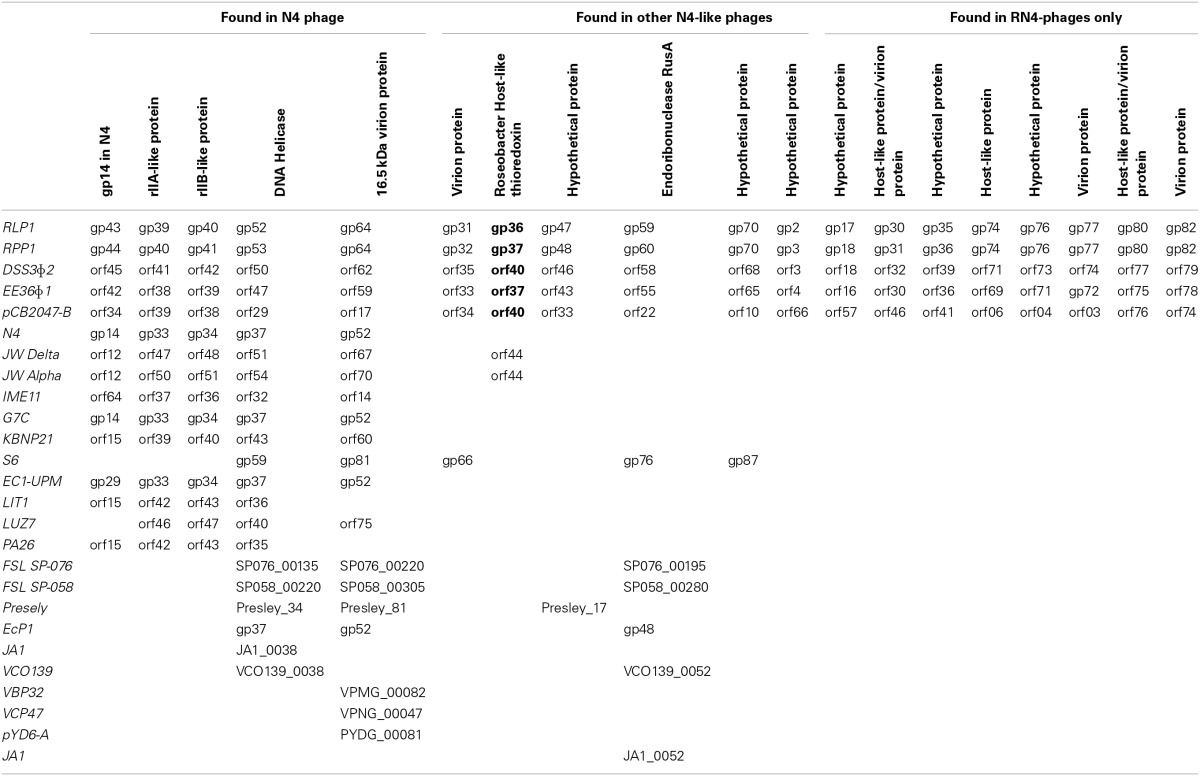
**Roseobacter phage genes**.

Homologs were identified by BLASTp searches and % identity at the nucleotide level calculated using ClustalW pairwise analysis. Genes in bold encode host-like AMGs.

The properties and genome sequences of these two novel phages are remarkably similar even though they were isolated from samples obtained 7 years apart, from two locations in UK coastal waters, and they infect different hosts (one isolated from the Caribbean the other from the English Channel). The host strains of these highly similar phage are only moderately close relatives at 93.5% 16S rRNA gene identity, and in case of RLP1, even the closest relative (*Rsv mucosus*, 99% 16S rRNA gene identity with *Rsv*. Sp. 217) was not infected. Although relatively few lytic phages of Roseobacters had been reported previously, it is intriguing that five of the seven lytic roseophages are closely related N4-like phages suggesting that similar phages may be common in the marine environment.

### Phylogenetic analysis of N4-like core genes

Analysis of the 25 sequenced N4-like phages identified 14 core genes, examples of these genes in N4 are listed in Table 3 (see Supplementary Material Table 1 for full list). This number of core is genes is similar to the 12 that were found for podoviruses infecting marine *Synechococcus* and *Prochlorococcus* (Labrie et al., 2013), however, the environments and hosts of the N4-like phage in this study are more diverse. Of these core genes five have no known function (designated as gps 24, 25, 53, 55, 69 in N4), leaving only nine genes that have putative function that are core to N4-like phage. As might be expected these are involved in processes that all N4-like phage would undergo regardless of the host they infect including DNA replication and packaging (gps 45, 50 and 68), transcription (gp15 and gp16) and production of structural proteins (gps 54, 55, 56 and 59). Interestingly, the homolog of RNAP2 in the *Achromobacter* phages JWAlpha and JWDelta has been divided into two parts due to the insertion of a 186 amino acid CDS similar to gp8 from *Celetribacter* phage P12053L (Wittmann et al., 2014). In N4, middle gene products are transcribed by a heterodimeric RNA polymerase the subunits of which are encoded by genes RNAP1 and RNAP2 (Willis et al., 2002). Though it is not clear if the RNAP2 homolog is functional in JWAlpha and JWDelta, we believe that the function of the gene product is essential and hence warrants its inclusion in the list of core genes.

**Table 3:** **Conserved core genes of the N4-like phage genus**.

**Gene in N4**	**Gene description**
15	RNAP1 (Transcriptional control)
16	RNAP2 (Transcriptional control)
24	Unknown
25	vWFA domain
39	DNA polymerase (DNA metabolism/replication)
45	SSB (DNA metabolism/replication)
50	vRNAP (DNA metabolism/replication)
53	Unknown
54	Structural protein (Structural)
55	Unknown
56	Major coat protein (Structural)
59	94 kDa portal protein (Structural)
68	Terminase, large subunit
69	Unknown

Homologs were identified using OrthoMCL which computes reciprocal best blast hit. An e-value cutoff of 1e-6 and I = 1.5 was used to identify the 14 core genes in the 25 publically available N4-like phage genomes.

Gene order of the core genes is largely conserved across all N4-like phage isolates (Figure 4) with unique/clade-specific genes tending to be toward the ends of the genomes. The insertion of genes specific to a subset of phage such as the RN4 phages also occurs at conserved positions as can be seen for *rnr* and *trx* (Figure 4). The high degree of synteny of the core genes involved in control of gene expression, DNA replication and structural proteins of 25 N4-like phages suggests that a stable association within each core module has been formed; conversely the areas between the blocks of core genes are likely hot-spots for recombination.

**Figure 5:**
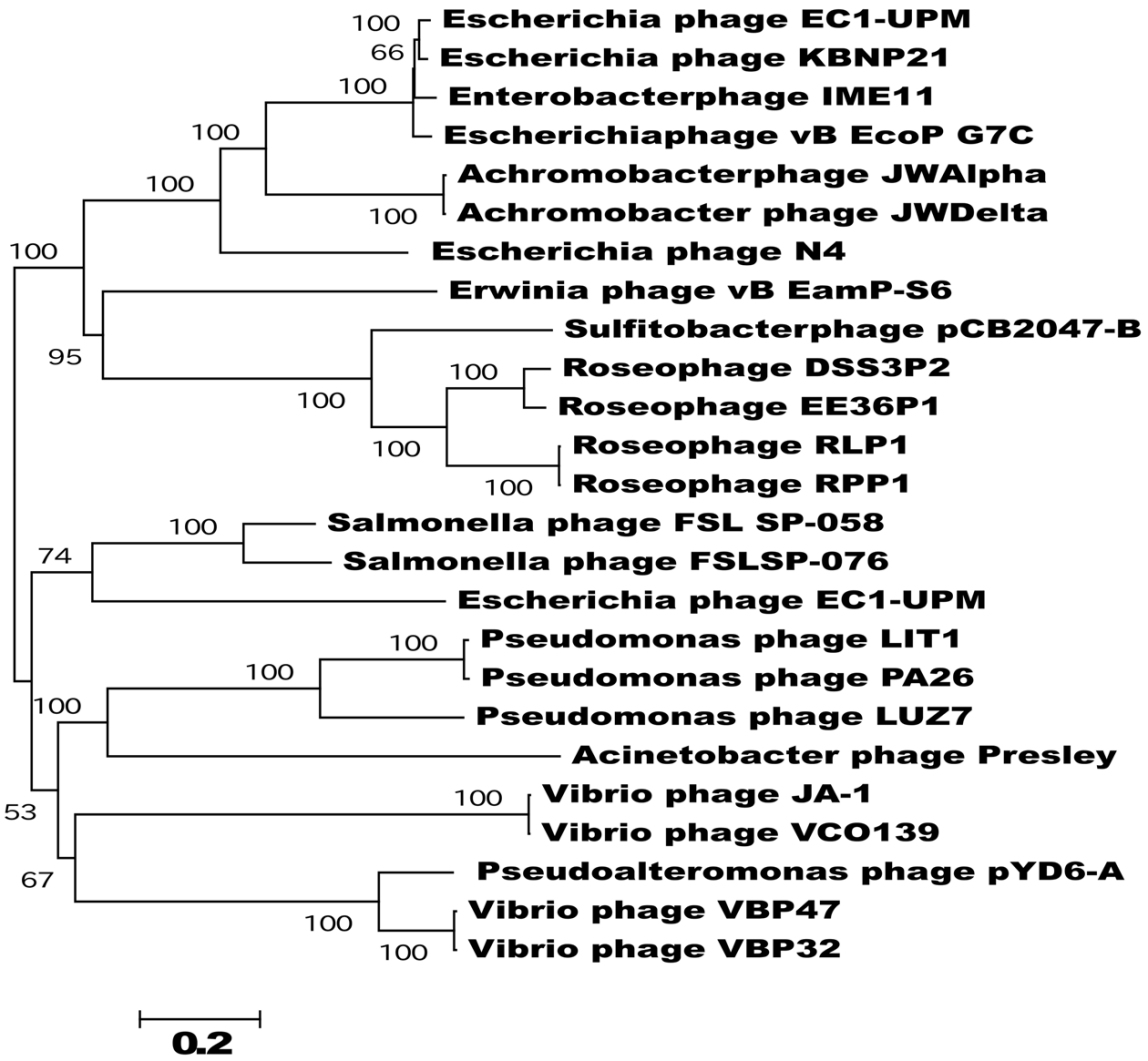
**Phylogram of concatenated core genes of the 25 sequenced N4-like phages**. The neighbor-joining tree was based on a ClustalW alignment of the concatenated core genes amino acid sequences; bootstrap values were based on 1000 replicates. Apart from Escherichia phage EC1-UPM, N4-like phages that infect closely related hosts cluster together on well supported branches. The tree is rooted at mid-point and branches with less than 50% bootstrap replicates were collapsed; scale bar indicate expected changes per site.

Phylogenetic analysis of the N4-like phages based on an alignment of concatenated core gene products showed that, with the exception of *Escherichia* phage EC1-UPM, phages that infect closely related hosts cluster together on well supported branches (Figure 5). For example, the five RN4-phages which infect marine Alphaproteobacteria, form a distinct clade away from their relatives that target gammaproteobacterial hosts. Furthermore, the two phages which infect *Roseovarius* species, RLP1 and RPP1, are further delineated from the other three RN4-phages; however, the phages EE36Φ1 and pCB2047-B that infect *Sulfitobacter* strains EE36 and 2047, respectively, did not form a distinct subclade. Overall the phylogeny based on concatenated core genes is concordant to that previously reported by Wittman et al. based on the proteomes of 24 N4-like phages (Wittmann et al., 2014). The delineation of N4 phage into clades that infect specific hosts suggests that all N4 phage shared a common ancestor and have since specialized to infect a particular group of hosts.

### Comparative analysis of RN4 phages pCB2047-B, DSS3Φ2, EE36Φ1, RLP1 and RPP1

Analysis of the five RN4-phages identified 33 conserved CDSs of which 14 are N4 core genes, five have homologs in N4 phage, five are found in other N4-like phages and nine are exclusive to the RN4 phages (Table 2). Interestingly one of the conserved RN4 phage genes, gp37 (in RPP1), is a host-like metabolic gene (known as auxiliary metabolic genes, AMGs; highlighted in bold in Table 2). Gp37 encodes a thioredoxin which has also been found in the T7-like Roseophage SIO1 (Rohwer et al., 2000). A homolog of this gene is also found in phages JWAlpha and JWDelta which were isolated from waste water treatment plants. It is interesting to note that whilst these phages infect *Achromobacter xylosoxidans*, a nosocomial pathogen widely distributed in the natural environment (Wittmann et al., 2014), other members of the *Achromobacter* genus are found in freshwater and marine environments (Brenner et al., 2005).

Phages DSS3Φ 2, EE36Φ 1, RLP1 and RPP1 share a further 22 CDSs (Supplementary Material Table 2) one of which, gp51 (in RPP1), is another AMG. RPP1 gp51 encodes a class II ribonucleoside diphosphate reductase (*rnr*). A previous study by Dwivedi et al., showed that the *rnr* genes in DSS3Φ 2 and EE36Φ 1 cluster together, with their bacterial host(s) forming a sister group (Dwivedi et al., 2013). A similar analysis using *trx* from the five RN4 phages, showed no clear relationship between phage and host genes (Supplementary Material Figure 2).

The presence of the AMG *trx* in the five RN4 phages is likely to represent an adaptation to the marine environment as it is common to all N4-like phages that infect marine bacteria (Figure 4). Thioredoxin-encoding genes can also be found in T7-like phages though it is also more common in viruses from the marine environment e.g., SIO1 and P60, than in enteric phages (Zhao et al., 2009). What the function of this gene might be is unclear; in bacteriophage T7 there is an increased rate of processing when thioredoxin binds to T7 DNA polymerase (Huber et al., 1987). However, whilst *trx* is found in other marine phages it is not clear if it serves the same function as found in T7 as the correct domain required for thioredoxin to bind may not be present (Hardies et al., 2003). Thioredoxin is known to have many other roles, one of which is a hydrogen donor to ribonucleotide reductase. This is possibly the most parsimonious function for *trx*, as four out of five RN4 phage also carry the *rnr* gene encoding for a ribonucleotide reductase. With *rnr* commonly found in other marine phage (Angly et al., 2006) it is thought to provide a mechanism of scavenging ribonucleotides in the oligotrophic marine environment (Sullivan et al., 2005). Therefore, it could be speculated for RN4 phages ribonuclease reductase is expressed to replicate the function of the host gene and the phage encoded thioredoxin acts in co-ordination as specific hydrogen donor, in a similar fashion that occurs in T4 (Holmgren, 1989).

### Identification of a second structural module in RPP1 phage

We identified, using mass spectrometry, 13 structural proteins in the mature RPP1/RLP1 virions (Table 4, Supplementary Material Figure 3) including five which have been identified as N4 virion proteins (gps 52, 54, 56, 59, and 67 in N4 phage/ gps 64, 66, 68, 71, and 77 in RPP1). Nine of the identified structural proteins in RPP1/RLP1 (gps 63, 64, 66, 68, 71 77, 80, 81, and 82 in RPP1) are likely “late” gene products inferred through synteny with N4 phage and their localization after the vRNAP gene and other late genes in N4 (Kazmierczak and Rothman-Denes, 2005). The remaining four (gps 25, 28, 31, and 32 in RPP1) are located near the N4 homologs of gp24 and 25 which in the Enterobacter phage N4 are middle gene transcripts (Kazmierczak and Rothman-Denes, 2005). This suggests there is a second structural module (SSM) in RPP1 which is expressed during the mid-phase of infection. Ceyssens et al. (2010) also identified a similar additional cluster of structural genes not expressed with the late genes in *Pseudomonas* phage LIT1 (Ceyssens et al., 2010). BLASTp analysis shows that the RPP1 gp32 gene product (a 650 aa protein) shares similarity with gp230 in *Pseudomonas* myovrius 201Φ 2-1, which is a fusion of homologs of Φ KZ gp145 and gp146, both tail proteins. Interestingly, genes within the second structural cluster in LIT1 (gps 48–56) have strong similarity to *Pseudomonas aeruginosa* prophage proteins and tail proteins from other *Podoviridae* (Ceyssens et al., 2010). Taken together, these observations suggest that the additional structural module encodes for and/or is associated with virion tail protein(s) production.

**Table 4:** **Genes encoding virion proteins in RLP1 and RPP1 identified by mass spectrometry**.

**Gene encoding identified virion protein**		**Homologous genes in RN4-like phages**			**Homologous gene in other N4-like phages**						**Comments**
**RPP1**	**RLP1**	**EE36φ1**	**DSS3φ2**	**pC2047-B**	**N4**	**JWDelta**	**JWAlpha**	**S6**	**LIT1**	**φJA1**	
gp25		gp23	gp25	–	–	–	–	–	–	–	1 putative chaperone domain
gp28		gp28	gp30	–	–	–	–	–	–	–	Many phage hypothetical protein homologs, 2 putative protein transport domains
gp31		gp30	gp32	SUFG000_46	–	–	–	–	–	–	Host-like protein
gp32		gp33	gp33	SUFG000_43	–	–	–	–	–	–	Abundant phage virion protein in phage 201Φ 2-1,10 putative domain of extracellular low-density lipoprotein receptor, 3 putative hydrolase, tail associated lysozyme in T4 domains
gp63	gp63	gp58	gp61	SUFG000_18	–	–	–	–	–	–	Possible similarity to C-terminal sequence of Roseophage SI01 gp24, hydrolase domain (residues 215–310)
gp64		gp59	gp62	SUFG000_16	gp52	gp67	gp70	gp81	gp73	gp55	16.5 kDa protein. Approx 41 copies/virion^*^
	gp66	gp61	gp65	SUFG000_15	gp54	gp66	gp69	gp50	gp40	gp54	Approx. 30 copies/virion^*^
gp68	gp68	gp63	gp66	SUFG000_13	gp56	gp71	gp74	gp85	gp77	gp59	Major capsid protein, approx 534 copies/virion^*^
gp71	gp71	gp66	gp69	SUFG000_9	gp59	gp74	gp77	gp55	gp50	gp362	94 kDa portal protein, approx 14 copies/virion^*^
gp77	gp77	gp72	gp74	SUFG000_3	gp67	–	–	–	–	–	30 kDa protein, approx 10 copies/virion^*^
gp80	gp80	gp75	gp77	SUFG000_76	–	–	–	–	–		Host-like protein, 10 predicted β-strands
gp81		gp76	gp78	–	–	–	–	–	–	–	No recognized protein domains
gp82	gp82	gp78	gp79	SUFG000_74	–	–	–	–	–	–	No recognized protein domains

* indicates values taken from Choi et al. (2008).

Proteins were separated on a 10–20% gradient SDS-PAGE. Bands of interest were excised, digested by trypsin and analyzed by nanoLC-ESI-MS/MS. Virion proteins identified are listed alongside the homologous genes found in other N4-like phages.

The gene products 25 and 28 in RPP1 found in the tail protein-linked SSM contain protein chaperone-like domains which could be associated with the translocation of the unfolded/semi-folded vRNAP out of the virion head into the host cell during initial infection. This is required as the virion polymerase is relatively large, 382.5 kDa, whilst the narrowest section of the tail tube in N4 is only 25 Å in diameter (Choi et al., 2008).

The location of these additional structural genes (upstream of the N4 gp45 homolog encoding an ssDNA-binding protein which activates transcription of late phage genes) suggests they are “middle” genes, but the advantage of expressing such proteins prior to the capsid genes is not yet clear. It may point to a gene regulation requirement and/or a possibility that tail proteins require maturation prior to assembly on the virion. In general, the constituent parts of phage virion particles (heads, tails and tail fibers) are made separately via subassembly pathways rather than a single linear pathway. Upon completion of the virion segment, the heads and tails combine first, forming complexes that are visible by electron microscopy, then the distal tail fibers are added (Campbell, 2007). It is possible that the assembly of the structurally complex tail portion of the virion may involve multiple steps and requires the assistance of helper proteins whilst the head is relatively simple to construct. Consequently, there might be an advantage in expressing some tail structural genes earlier than the genes coding for head, portal and other tail fiber genes.

Of the 13 structural proteins identified in RPP1/RLP1, 10 are conserved in all the sequenced RN4 phages. These include gps 31 and 32 (in RPP1) from the SSM. Interestingly whilst gp31 is only shared by the RN4 phages, a homolog of gp32 is also found in *Erwinia* phage S6 (Born et al., 2011) as gp66. The aforementioned gps 25 and 28 (in RPP1) are only found in phages DSS3Φ 2, EE36Φ 1 and RLP1 suggesting this module could be a determinant of host specificity whilst gene product 81 is only found in RLP1 and RPP1.

### Environmental distribution of RN4-like phages

**Figure 6:**
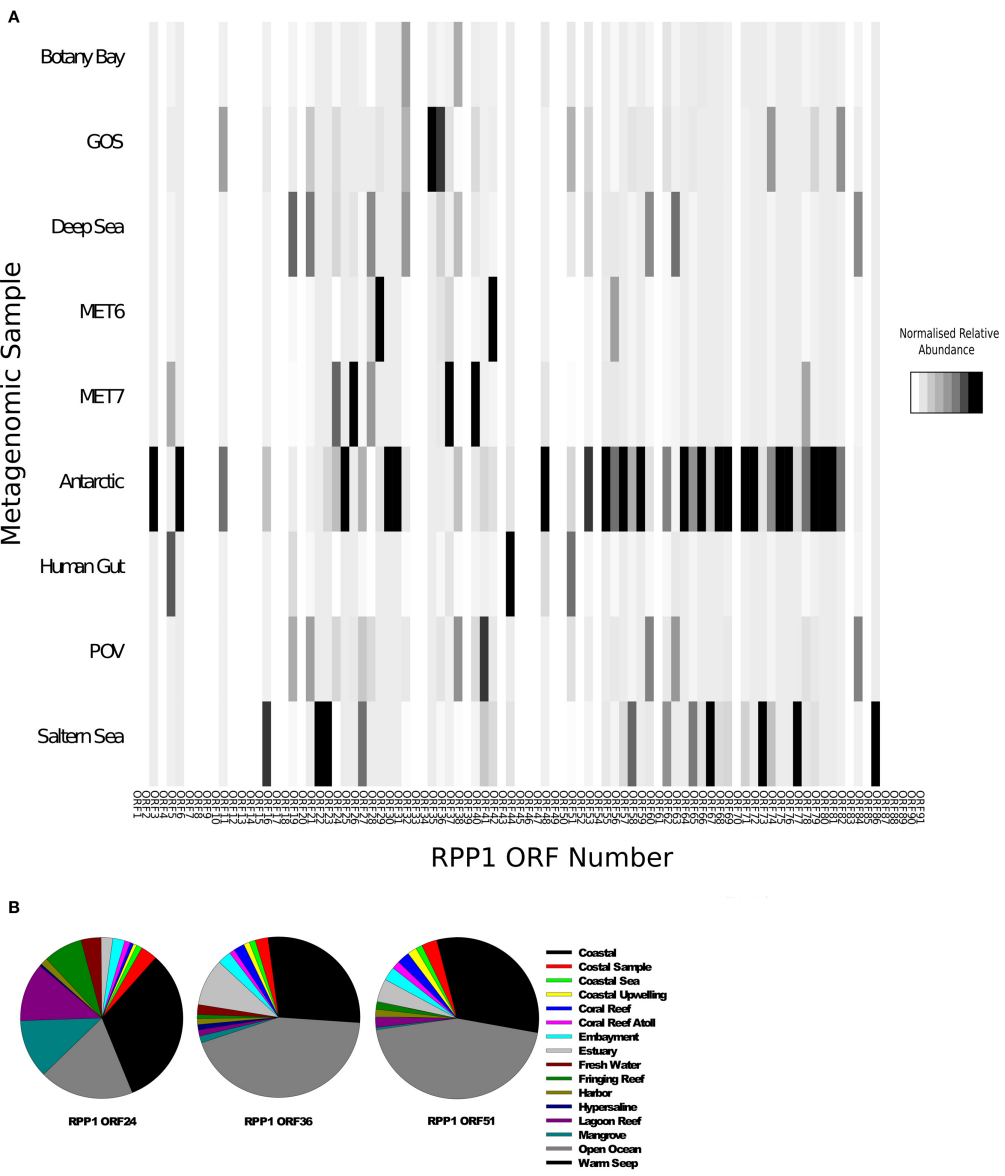
**Relative abundance of RN4-like phage genes in various metagenomes**. **(A)** Heatmap of the normalized relative abundance of RPP1 ORFs identified in the Global Ocean Survey (GOS), Botany Bay, Deep sea, Lake Pavin (MET7), Lake Bourget (MET6), Antarctic, human gut and the Saltern metagenomes. **(B)** Normalized relative abundance of ORFs 24, 38, and 51 in the stations sampled by the Global Ocean Survey. Samples were grouped together based on the environment of the station as previously defined by Venter et al. (2004).

Using all the CDS sequences in RPP1 as blast query against a range of environmental metagenomic datasets downloaded from CAMERA (Sun et al., 2011) we searched for RN4-like phage sequences. The number of hits were normalized for database size and gene size to allow comparison between metagenomes (see Materials and Methods for further details). Previous searches of Global Ocean Survey (GOS) metagenomic data using RN4 polymerase genes as well as the other N4-like genes as query sequences suggested that N4-like phage infecting Roseobacters are mainly found in coastal areas and may be rare in open ocean environments (Zhao et al., 2009). We found homologs of CDSs from RN4 phages are widespread in a number of environments (Figure 6A) with the highest frequency of counts in samples from the Antarctic, Saltern Sea and GOS metagenomes. As expected, given the known distribution of members of the Roseobacter lineage, we found very low detection rates in the metagenomes from freshwater lakes (MET6, MET7).

A more detailed analysis of the distribution of hits found in the GOS metagenome was carried out based on the previously defined environments as reported by the Sorcerer II GOS expedition (Rusch et al., 2007). The distribution of three RPP1-like genes for each GOS sampling site was carried out using the three most abundant gene sequences identified previously, ORFs 24, 38, and 51, as queries. A large proportion of matches were found in locations characterized as a coastal environment (Figure 6B); this would be expected based on the distribution of Roseobacter hosts in costal environments. However, for some genes—ORF36 and ORF51, a higher percentage of hits were found in samples from open ocean environments (Figure 6B), thus suggesting that there are more RN4-like phage, and their corresponding hosts, present in the open ocean environment than previously thought. However, this finding should be considered with caution as we presume the hosts of these phages belong to the Roseobacter lineage. There is the possibility that these are not RN4 phages and instead belong to a different family of podoviruses that infect another group of bacteria which have not yet been cultured and/or had their genome sequenced.

### Evolution of the N4-like phage genus and beyond

The genome arrangement of core and variable genes within this phage genus bears striking similarity to the T4 superfamily in which the genomes have been defined as bipartite (Krisch and Comeau, 2008); a conserved core comprised of the minimal essential genes required for viral multiplication and a larger, highly variable set of facultative genes which collectively create an optimal environment, particular to that host, to enable successful infection. However, in the T4 superfamily most of the “core T4” genes encode either virus replication functions or virion structural components. As N4 has such an unusual gene expression mechanism (Kazmierczak and Rothman-Denes, 2005), it is perhaps not surprising to find genes involved in transcription control to be conserved, such as the three RNA polymerase genes and the single-stranded DNA-binding protein involved in late gene expression.

In the T4 superfamily, the number of core genes varies according to the subset of phages considered. For example, there are 75 common core genes when “true” T-even (T4), pseudo T-even (RB49) and schizo T-even (Aeh1) are compared (Sullivan et al., 2005; Clokie et al., 2010), but this falls to 38 when the cyanophages are included (Millard et al., 2009; Sullivan et al., 2010). With the N4-like phages, the subdivisions below genus level are not as clear but it appears that core genes from phages which infect closely related hosts bear more similarity to each other than those from evolutionary distant hosts as seen by the clustering of the RN4, *Pseudomonas*, Enterobacter/*Escherichia*, and Vibrio phages (Figure 4). In addition to vertical gene transfer, horizontal gene exchanges could have occurred from both phage (*Pseudomonas* tail proteins and the *trx* gene) and host (Roseobacter host-like proteins e.g., *rnr*) sources.

Phage biologists have long debated as to whether or not phage genera actually exist or if instead there is a continuum of phage genes in which all tailed-phages dip into, to find a “best-fit” genome. The mosaic model proposed by Hendrix et al., poses the best compromise to this problem (Hendrix et al., 1999), proposing that early phages have exchanged large chunks of genetic information prior to the demarcation of the now accepted supergroups. Fine tuning of host/environmental specific genes between close relatives then followed, the consequence of which are phages with genomes created from a mixture of vertical and horizontal gene transfer events. The results from this study fit in well with this theory. The 14 core genes, which encode and control general infectivity, appear to be derived from ancient phages thus accounting for the homology and gene synteny found in the terrestrial and marine phages, whilst the plastic periphery is comprised of genes such as *rnr, trx* and the tail/tail fiber structural proteins which provide environmental adaptations and determine the host range. However, further analyses are required to determine if the latter set of genes were horizontally or vertically acquired. Such studies and characterization of more N4-like phages, in particular those from the marine environment, will allow further population genetic type analyses of this diverse phage group.

### Conflict of interest statement

The authors declare that the research was conducted in the absence of any commercial or financial relationships that could be construed as a potential conflict of interest.
