# Supplementary material for: Comparative genomics defines the core genome of the growing N4-like phage genus and identifies N4-like Roseophage specific genes
Source: Front Microbiol. 2014 Oct 10;5:506. doi: 10.3389/fmicb.2014.00506 (PMC4193335; doi:10.3389/fmicb.2014.00506)
Supplement: Supplementary file 1 [file DataSheet1.DOCX]

***Supplementary Material***

**Comparative genomics defines the core genome of the growing N4-like phage genus and identifies Roseophage specific genes**

**Jacqueline Z-M Chan^1^*, Andrew D Millard^2^, Nicholas H Mann^2^, Hendrik Schäfer^3^**

^1^Laboratory Oxford Gene Technologies, Begbroke Sciences Park, Begbroke Hill, Woodstock Road, Oxfordshire, OX5 1PF, UK

^2^Division of Microbiology and Infection, Warwick Medical School, University of Warwick, Coventry, CV4 7AL, UK

^3^Department of Life Sciences, University of Warwick, Coventry CV4 7AL, UK

*** Correspondence:** Jacqueline Chan, Laboratory Oxford Gene Technologies, Begbroke Sciences Park, Begbroke Hill, Woodstock Road, Oxfordshire, OX5 1PF, UK

[Jackie.chan@ogt.com](mailto:Jackie.chan@ogt.com)

1. **Supplementary Table 1. Core genes of the N4-like genus.**

Homologous genes were identified using OrthoMCL (Li et al., 2003) which computes a bidirectional best hit serach (evalue cutoff = 1e^-6^, I = 1.5) The initial database was construction from the amino acid sequence of all predicted proteins extracted from the 25 publically available Genbank files. *indicates RNAP2 has been split into two CDSs in *Achromobacter* phages JWAlpha and JWDelta.

|  | *RNAP1* | *RNAP2* | *gp24* | *gp25* | *DNAP/gp39* | *ssb/gp45* | *vRNAP* | *gp53* | *gp54* | *gp55* | *MCP/gp56* | *94 kDa protein/*  *gp59* | *gp68* | *gp69* |
| --- | --- | --- | --- | --- | --- | --- | --- | --- | --- | --- | --- | --- | --- | --- |
| N4 | AAL71577 | AAL71578 | ABK54393 | ABK54394 | ABK54408 | AAA86389 | AAO24831 | AAO24828 | AAO24827 | AAO24826 | AAO24825 | ABK54420 | ABK54429 | ABK54430 |
| RLP1 | CBW46999 | CBW47009 | CBW47012 | CBW47014 | CBW47048 | CBW47052 | CBW47056 | CBW47059 | CBW47060 | CBW47061 | CBW47062 | CBW47065 | CBW47072 | CBW47073 |
| RPP1 | CBX87936 | CBX87946 | CBX87949 | CBX87951 | CBX87985 | CBX87989 | CBX87992 | CBX87995 | CBX87996 | CBX87997 | CBX87998 | CBX88001 | CBX88008 | CBX88009 |
| DSS3ɸ2 | ACL81274 | ACL81284 | ACL81288 | ACL81290 | ACL81320 | ACL81325 | ACL81328 | ACL81331 | ACL81332 | ACL81333 | ACL81334 | ACL81337 | ACL81343 | ACL81344 |
| EE36ɸ1 | ACL81356 | ACL81364 | ACL81367 | ACL81369 | ACL81397 | ACL81403 | ACL81406 | ACL81409 | ACL81410 | ACL81411 | ACL81412 | ACL81415 | ACL81422 | ACL81423 |
| LIT1 | CAZ66275 | CAZ66279 | CAZ66289 | CAZ66291 | CAZ66294 | CAZ66318 | CAZ66327 | CAZ66330 | CAZ66331 | CAZ66332 | CAZ66333 | CAZ66336 | CAZ66340 | CAZ66341 |
| LUZ7 | CAZ66161 | CAZ66163 | CAZ66178 | CAZ66180 | CAZ66183 | CAZ66206 | CAZ66214 | CAZ66217 | CAZ66218 | CAZ66219 | CAZ66220 | CAZ66223 | CAZ66226 | CAZ66227 |
| S6 | AEJ81547 | AEJ81550 | AEJ81554 | AEJ81555 | AEJ81582 | AEJ81593 | AEJ81598 | AEJ81601 | AEJ81602 | AEJ81603 | AEJ81604 | AEJ81607 | AEJ81617 | AEJ81618 |
| KBNP21 | AFR51968 | AFR51970 | AFR51978 | AFR51979 | AFR51996 | AFR52003 | AFR52009 | AFR52012 | AFR52013 | AFR52014 | AFR52015 | AFR52018 | AFR52033 | AFR52026 |
| PA26 | AFO70518 | AFO70521 | AFO70532 | AFO70533 | AFO70536 | AFO70559 | AFO70568 | AFO70571 | AFO70572 | AFO70573 | AFO70574 | AFO70577 | AFO70581 | AFO70582 |
| G7C | AEL79622 | AEL79624 | AEL79630 | AEL79632 | AEL79649 | AEL79656 | AEL79661 | AEL79664 | AEL79665 | AEL79666 | AEL79667 | AEL79670 | AEL79678 | AEL79679 |
| IME11 | AFV29104 | AFV29104 | AFV29104 | AFV29104 | AFV29079 | AFV29073 | AFV29104 | AFV29104 | AFV29104 | AFV29060 | AFV29104 | AFV29056 | AFV29104 | AFV29104 |
| EC1-UPM | AGC31525 | AGC31526 | AGC31533 | AGC31534 | AGC31551 | AGC31559 | AGC31565 | AGC31568 | AGC31569 | AGC31570 | AGC31571 | AGC31574 | AGC31502 | AGC31503 |
| FSL SP-058 | YP_008239422 | YP_008239426 | YP_008239439 | YP_008239440 | YP_008239451 | YP_008239458 | YP_008239463 | YP_008239466 | YP_008239467 | YP_008239468 | YP_008239469 | YP_008239472 | YP_008239484 | YP_008239485 |
| FSL SP-076 | YP_008240153 | YP_008240156 | YP_008240168 | YP_008240169 | YP_008240179 | YP_008240186 | YP_008240191 | YP_008240194 | YP_008240195 | YP_008240196 | YP_008240197 | YP_008240200 | YP_008240212 | YP_008240213 |
| JA1 | AGI61764 | AGI61766 | AGI61788 | AGI61789 | AGI61793 | AGI61803 | AGI61805 | AGI61808 | AGI61809 | AGI61810 | AGI61811 | AGI61814 | AGI61819 | AGI61820 |
| Presely | AGY48085 | AGY48088 | AGY48098 | AGY48100 | AGY48104 | AGY48136 | AGY48146 | AGY48149 | AGY48150 | AGY48151 | AGY48152 | AGY48155 | AGY48157 | AGY48159 |
| VCO139 | AGI61840 | AGI61842 | AGI61865 | AGI61866 | AGI61870 | AGI61880 | AGI61882 | AGI61884 | AGI61885 | AGI61886 | AGI61887 | AGI61890 | AGI61894 | AGI61895 |
| JW Alpha | AHC93967 | AHC56533/  AHC56531* | AHC93986 | AHC93989 | AHC94009 | AHC94015 | AHC94021 | AHC94024 | AHC94025 | AHC94026 | AHC94027 | AHC94030 | AHC94037 | AHC94038 |
| JW Delta | AHC56530 | AHC93970/  AHC93968* | AHC56548 | AHC56551 | AHC56569 | AHC56575 | AHC56581 | AHC56584 | AHC56585 | AHC56586 | AHC56587 | AHC56590 | AHC56597 | AHC56598 |
| pCB2047-B | AGH07432 | AGH07425 | AGH07422 | AGH07420 | AGH07396 | AGH07395 | AGH07392 | AGH07388 | AGH07387 | AGH07386 | AGH07385 | AGH07382 | AGH07375 | AGH07374 |
| EcP1 | ADU79156 | ADU79161 | ADU79184 | ADU79185 | ADU79191 | ADU79198 | ADU79201 | ADU79204 | ADU79205 | ADU79206 | ADU79207 | ADU79210 | ADU79217 | ADU79218 |
| pYD6-A | YP_007674232 | YP_007674237 | YP_007674251 | YP_007674261 | YP_007674265 | YP_007674283 | YP_007674286 | YP_007674289 | YP_007674290 | YP_007674291 | YP_007674292 | YP_007674295 | YP_007674305 | YP_007674306 |
| VBP32 | AGH57167 | AGH57158 | AGH57145 | AGH57248 | AGH57244 | AGH57227 | AGH57223 | AGH57220 | AGH57219 | AGH57218 | AGH57217 | AGH57213 | AGH57204 | AGH57201 |
| VCP47 | AGH57124 | AGH57133 | AGH57033 | AGH57044 | AGH57048 | AGH57065 | AGH57069 | AGH57072 | AGH57073 | AGH57074 | AGH57075 | AGH57079 | AGH57088 | AGH57091 |

1. **Supplementary Figure 1.**


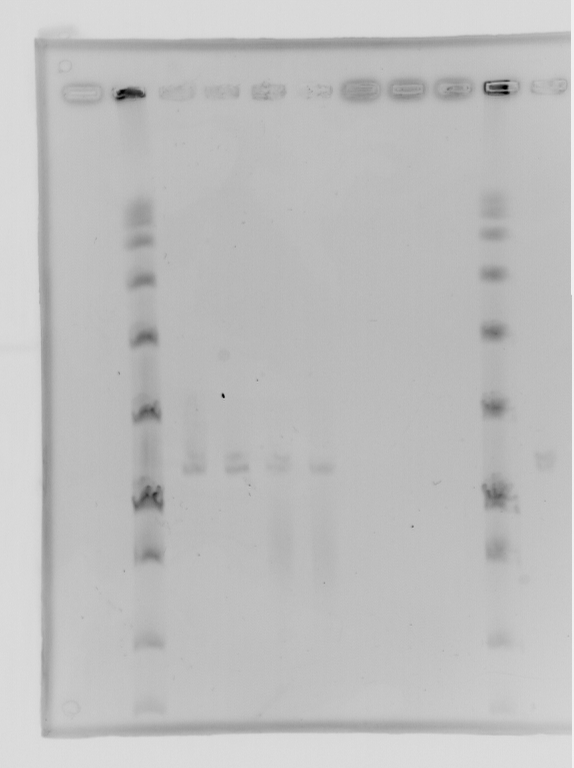


**kb M 1 2 3 4**

97

145

48.5

23.1

Figure 1. Pulsed field gel electrophoresis of purified RLP1 and RPP1 genomic DNA. Phage DNA was obtained by in plug digestion of high titre (10^5^ – 10^6^ PFU/ml) CsCl purified phage. Samples were run in a 1 % (w/v) PFGE grade agarose gel. M: Marker, sizes given in kb; lanes 1 & 2: RLP1 genomic DNA, lanes 3 & 5: RPP1 genomic DNA

1. **Supplementary Figure 2.**

**
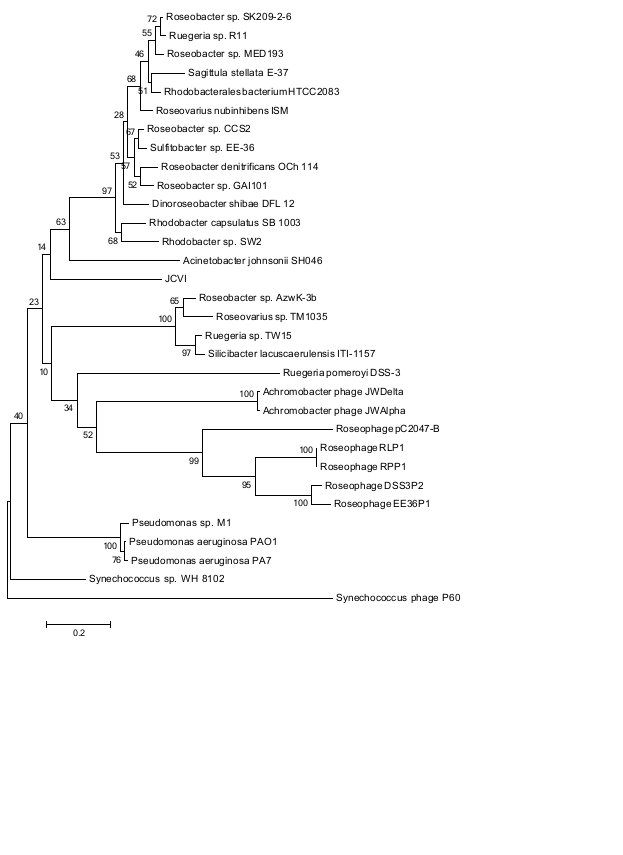
**

**Figure 2. Phylogenetic analysis of *trx* genes from the five RN4 phages, *Achromobacter* phages JWAlpha and Delta, other marine viruses and sequenced Roseobacter species.** The tree was inferred using the neighbor-joined method (Saitou and Nei, 1987). Phylogenetic analyses were conducted in MEGA6. There appears to be no clear relationship between the RN4 phages and host genes.

1. **Supplementary Figure 3.**


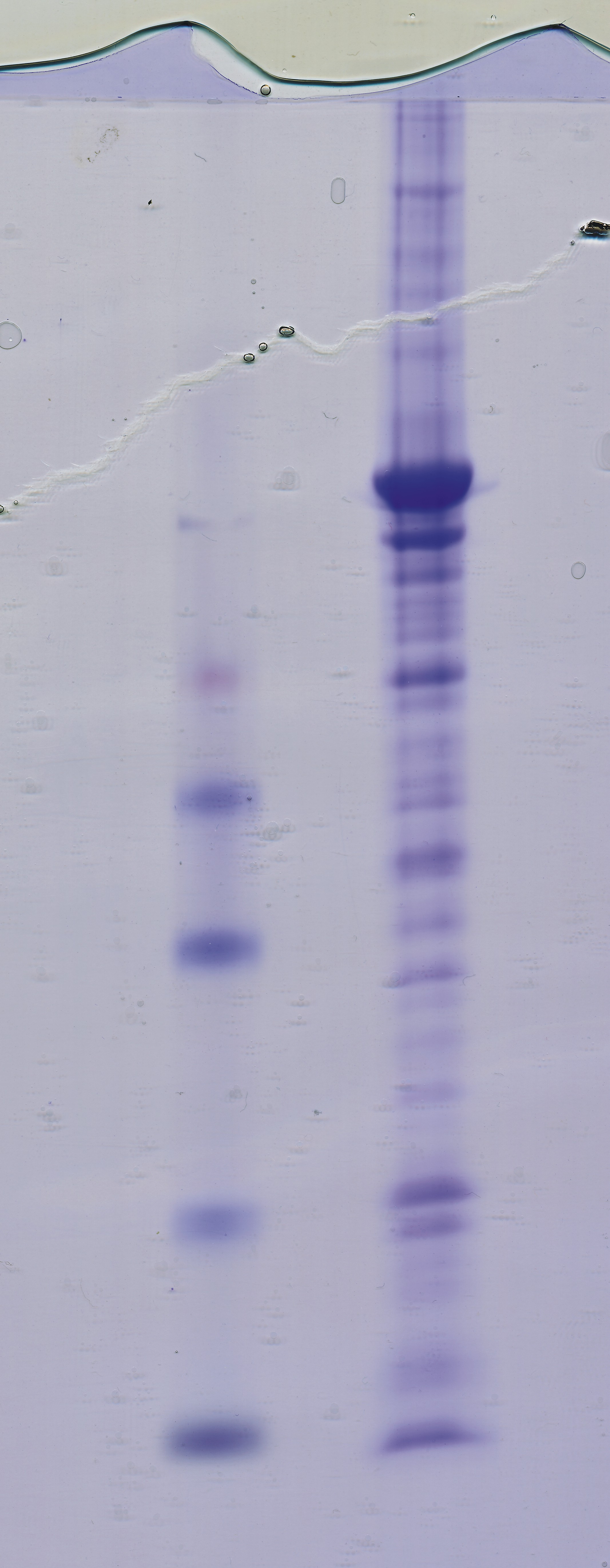

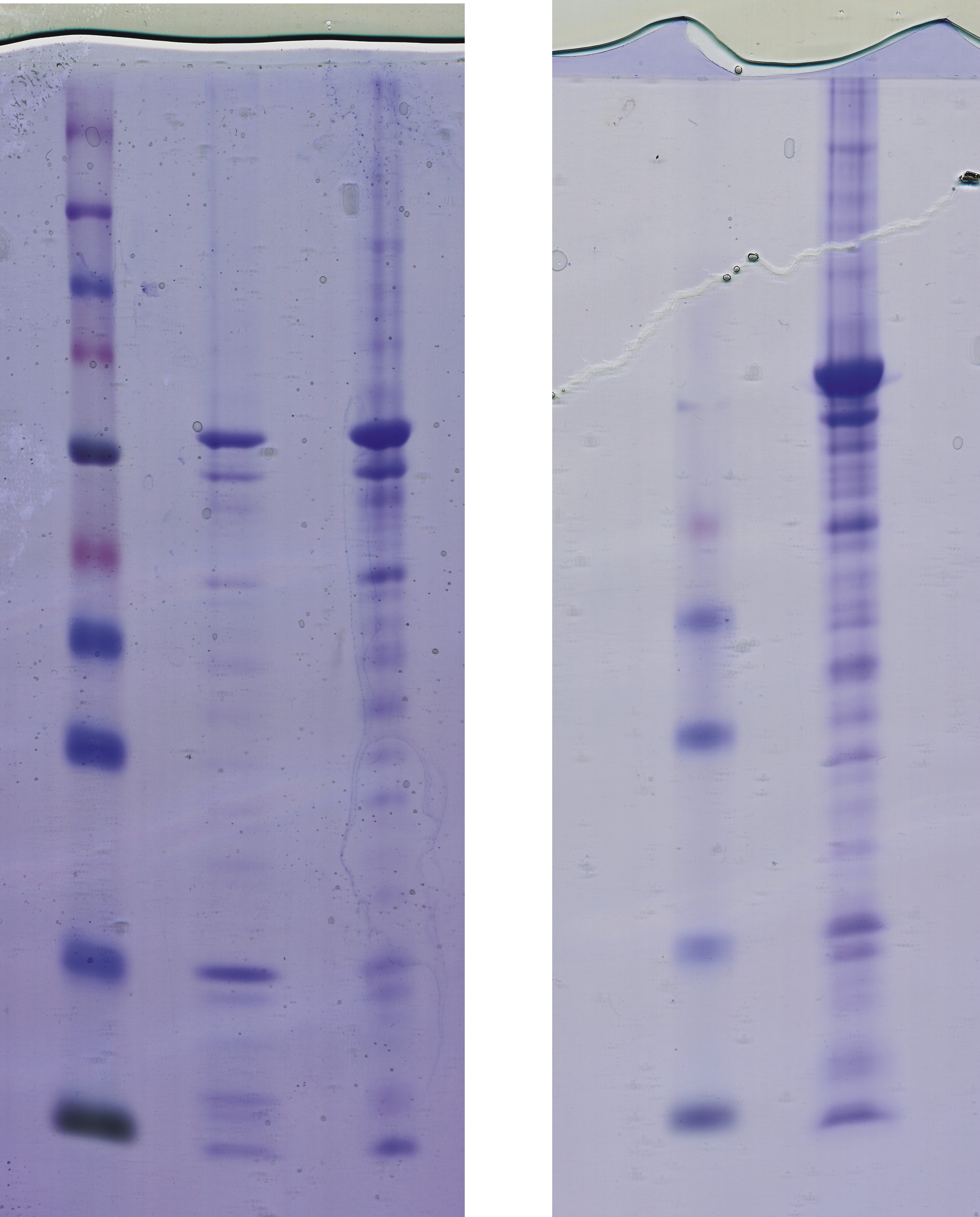

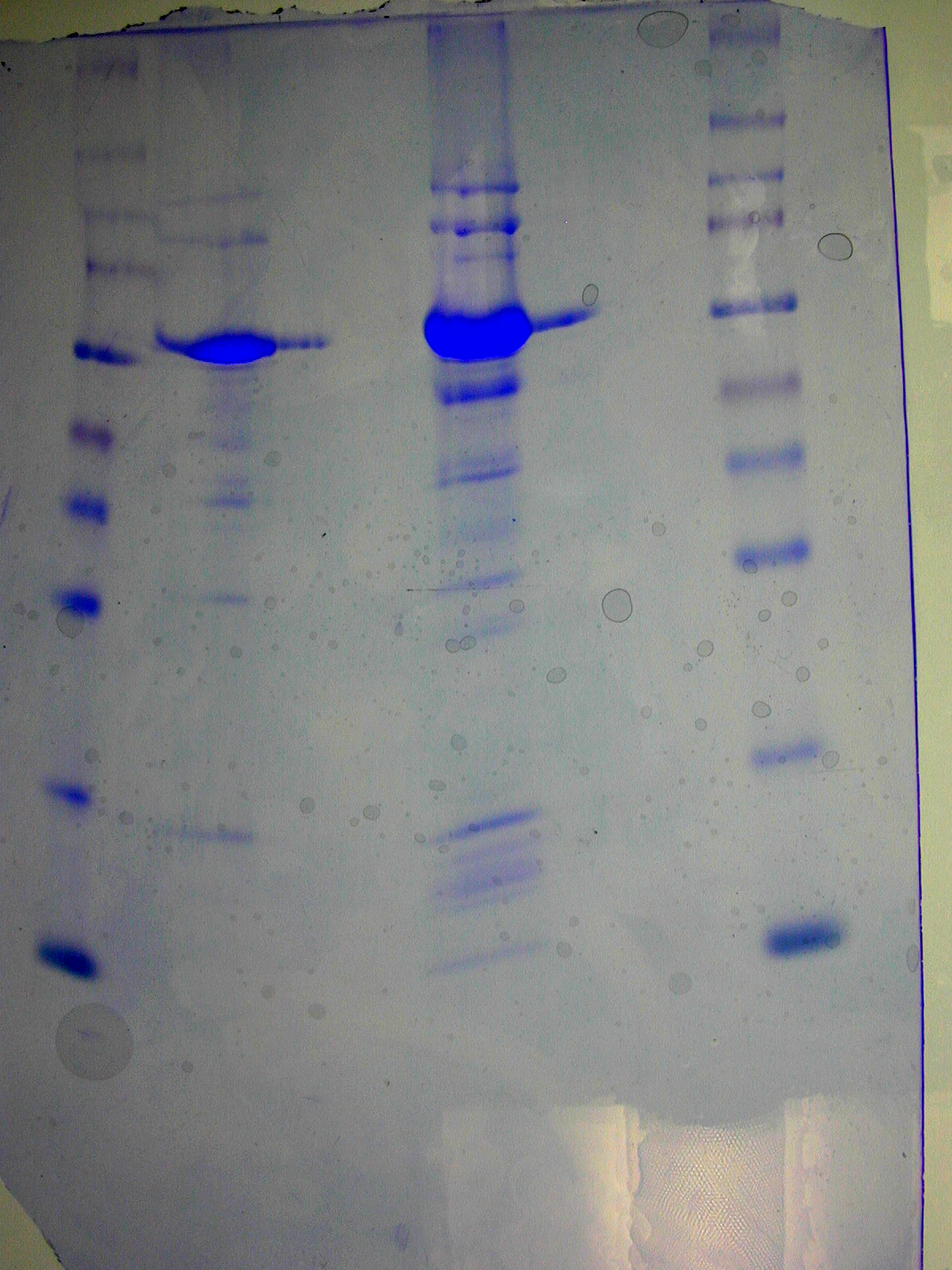

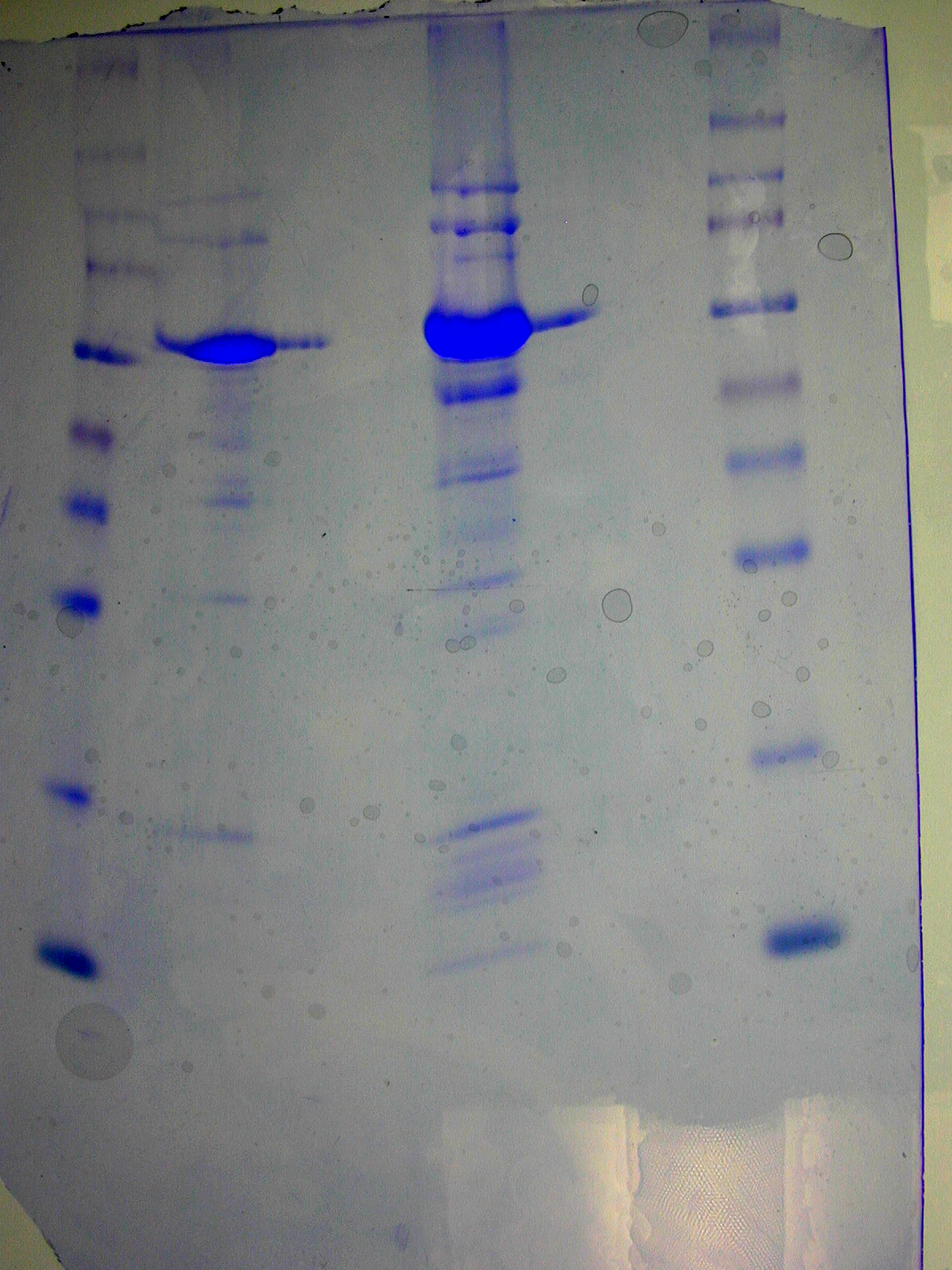


**M**

**M**

**RLP1**

**RPP1**

**260**

**135**

**95**

**72**

**52**

**42**

**34**

**26**

**17**

**10**

**260**

**135**

**95**

**72**

**52**

**42**

**34**

**26**

**17**

**10**

**B1**

**B2**

**B3**

**B4**

**B6**

**B7, 8, 9**

**B10**

**B11**

**B12**

**B13**

**B14**

**BP15**

**B16**

**B17**

**B18, 19**

**B20**

**B22**

**B23**

**B24**

**B25**

**B26**

**B27**

**B28**

**B29**

**B30, 31**

**B32**

**B34**

**B33**

**B5**

**kDa**

**kDa**

| **Band** | | **Protein** | **No. of peptides** | **Approx. mass/ kDa** |
| --- | --- | --- | --- | --- |
|  |  |  |  |  |
| **RLP1** | | | | |
| B1 | gp63 | | 5 | 7.5 |
|  | gp68 | | 4 | 51.4 |
| B2 | gp68 | | 6 | 5.1 |
|  | gp80 | | 4 | 10.4 |
|  | gp63 | | 2 | 75 |
| B3 | gp63 | | 8 | 7.5 |
|  | gp68 | | 5 | 51.4 |
|  | gp66 | | 2 | 43.7 |
|  | host contamination, putative lipoprotein | | 2 | 14.7 |
| B4 | gp68 | | 8 | 51.4 |
|  | gp63 | | 7 | 7.5 |
| B5 | gp68 | | 12 | 51.4 |
|  | gp63 | | 8 | 7.5 |
|  | host contamination, putative lipoprotein | | 2 | 14.9 |
| B6 | gp68 | | 10 | 51.4 |
|  | gp63 | | 4 | 7.5 |
| B7 | gp63 | | 10 | 7.5 |
|  | gp68 | | 7 | 51.4 |
|  | gp71 | | 7 | 88.9 |
| B8 | gp68 | | 8 | 51.4 |
|  | gp71 | | 5 | 88.9 |
|  | gp82 | | 3 | 35.9 |

| **Band** | **Protein** | **No. of peptides** | **Approx. mass/ kDa** |
| --- | --- | --- | --- |
|  |  |  |  |
| B9 | gp68 | 11 | 51.4 |
|  | gp77 | 3 | 25.1 |
|  | host contamination, putative lipoprotein | 2 | 36.8 |
|  | gp63 | 2 | 7.5 |
| B10 | gp68 | 12 | 51.4 |
|  | gp77 | 9 | 25.1 |
|  |  | 2 | 36.8 |
| B11 | gp68 | 11 | 51.4 |
|  | gp63 | 4 | 7.5 |
|  | gp71 | 4 | 88.9 |
|  | gp82 | 2 | 35.9 |
| B12 | gp68 | 10 | 51.4 |
|  | gp71 | 6 | 88.9 |
|  | gp82 | 6 | 35.9 |
|  | host contamination, putative lipoprotein | 2 | 36.8 |
|  | gp63 | 2 | 7.5 |
| B13 | gp68 | 13 | 51.4 |
|  | gp71 | 6 | 88.9 |
|  | host contamination, putative lipoprotein | 4 | 36.8 |
| B14 | gp71 | 13 | 88.9 |
|  | gp66 | 9 | 43.7 |
|  | gp68 | 7 | 51.4 |
|  | host contamination, putative lipoprotein | 4 | 36.8 |
|  | gp63 | 3 | 7.5 |

| **Band** | **Protein** | **No. of peptides** | **Approx. mass/ kDa** |
| --- | --- | --- | --- |
|  |  |  |  |
| B15 | gp66 | 13 | 43.7 |
|  | gp68 | 12 | 51.4 |
|  | gp71 | 6 | 88.9 |
|  | host contamination, putative lipoprotein | 2 | 36.8 |
|  | gp63 | 2 | 7.5 |
| B16 | gp68 | 14 | 51.4 |
|  | gp66 | 10 | 43.7 |
|  | gp63 | 9 | 7.5 |
|  | gp71 | 4 | 88.9 |
| B17 | gp71 | 17 | 88.9 |
|  | gp63 | 10 | 7.5 |
|  | gp68 | 10 | 51.4 |
| B18 | gp68 | 11 | 51.4 |
|  | gp63 | 7 | 7.5 |
|  | gp71 | 5 | 88.9 |
| B19 | gp63 | 21 | 7.5 |
|  | gp68 | 11 | 51.4 |
|  | gp71 | 8 | 88.9 |
|  | gp66 | 2 | 43.7 |
| B20 | gp68 | 15 | 51.4 |
|  | gp71 | 11 | 88.9 |
|  | gp63 | 3 | 7.5 |
| B21 | gp68 | 14 | 51.4 |
|  | gp71 | 6 | 88.9 |
|  | gp63 | 3 | 7.5 |

| **Band** | **Protein** | **No. of peptides** | **Approx. mass/ kDa** |
| --- | --- | --- | --- |
|  |  |  |  |
| **RPP1** | | | |
| B22 | gp71 | 25 | 88.9 |
|  | gp68 | 3 | 51.4 |
| B23 | gp63 | 22 | 75.1 |
|  | gp68 | 4 | 51.4 |
| B24 | gp63 | 7 | 75.1 |
|  | gp32 | 5 | 68.2 |
|  | gp63 | 1 | 75.1 |
| B25 | gp68 | 17 | 51.4 |
| B26 | gp68 | 9 | 51.4 |
| B27 | gp82 | 5 | 36 |
|  | gp68 | 2 | 51.4 |
| B28 | gp82 | 6 | 36 |
|  | gp68 | 4 | 51.4 |
| B29 | gp77 | 19 | 25.2 |
|  | gp68 | 3 | 51.4 |
| B30 | gp28 | 1 | 44.4 |
|  | gp68 | 1 | 51.4 |
| B31 | gp68 | 2 | 51.4 |
|  | gp81 | 1 | 22.3 |
| B32 | gp64 | 2 | 16.1 |
|  | gp25 | 1 | 17.2 |
| B33 | gp31 | 3 | 12.5 |
| B34 | gp80 | 2 | 10.4 |

**Supplementary figure 3. Identification of virion proteins of phages RLP1 and RPP1 by gradient SDS-PAGE.** Proteins, extracted by TCA precipitation from approximately 1012 double CsCl purified virions, were separated on a 10 – 20 % gradient SDS-polyacrylamide gel set at 100 V for 18 hours. Boxes indicate the bands excised for mass spectrometry. As the genomic information indicated that both phages were highly similar it was assumed that the phage proteins would be highly similar. As such duplicate bands present in both gels were only analysed once e.g. B25, the predicted major capsid protein was only analysed in the RPP1 protein gel. This was done to ensure the maximum coverage of bands present in both phages.

1. **References**

Li, L., Stoeckert, C. J., and Roos, D. S. (2003). OrthoMCL: Identification of Ortholog Groups for Eukaryotic Genomes. *Genome Res.* 13 , 2178–2189. doi:10.1101/gr.1224503 .

Saitou, N., and Nei, M. (1987). The neighbor-joining method: A new method for reconstructing phylogenetic trees. *Mol Biol Evol* 4, 406–425.
